# The effect of TGFβ1 on thermogenic markers is dependent on the degree of adipocyte differentiation

**DOI:** 10.1042/BSR20194262

**Published:** 2020-05-14

**Authors:** Babu R. Maharjan, Susan V. McLennan, Stephen M. Twigg, Paul F. Williams

**Affiliations:** 1 Greg Brown Diabetes and Endocrinology Laboratory, Sydney Medical School, University of Sydney, Sydney, NSW, Australia; 2 New South Wales Health Pathology, Australia; 3 Department of Endocrinology, Royal Prince Alfred Hospital, Sydney, Australia

**Keywords:** adipocyte, adipogenesis, stages of differentiation, TGFβ, thermogenesis

## Abstract

Transforming growth factor β (TGFβ) a multifunctional cytokine is known to regulate cell proliferation, differentiation, migration and survival. Although there is variable expression of modulators of TGFβ action during differentiation, a differential effect on fat cell metabolism at the different stages of adipocyte differentiation was unclear. In the present study, 3T3L1 cells were used as an *in vitro* model to study the effect of TGFβ on adipogenic and thermogenic markers at various stages of preadipocyte to mature adipocyte differentiation. As in our earlier studies on the effect of TGFβ on CEBP’s, we used a standard differentiation mix, and one with the addition of rosiglitazone. RhTGFβ1 was added to undifferentiated adipocytes (preadipocytes) and to adipocytes at day 0 (commitment stage) as well as day 10 (terminal differentiation). Cellular responses in terms of Pref1, PPARγ, TLE3, PGC1α, PRDM16, UCP1 and UCP2 mRNA levels and selected protein products, were determined. Increases in PPARγ, PRDM16, UCP1 and UCP2 mRNA and decreases in Pref1 are good indicators of successful differentiation. The early addition of rhTGFβ1 during commitment stage decreased PPARγ, PRDM16, TLE3, UCP1 and UCP2 mRNA and decreased PRDM16 protein consistent with our earlier report on the inhibition of CEBP’s by TGFβ and CCN2. The addition of rhTGFβ1 to mature adipocyte at day 10 increased UCP1 mRNA and increased PRDM16 and UCP1 proteins. In the present study, our results suggest that TGFβ1 added late enhances the thermogenic potential of mature cells and causes 3T3L1 cells to differentiate to resemble brown or beige rather than white adipose tissue.

## Introduction

Along with its known fibrogenic activity, TGFβ is a critical cytokine mediator of both insulin resistance and hepatic steatosis in obesity-induced metabolic diseases [1,2]. Circulating TGFβ levels are significantly elevated in obese humans, ob/ob and high-fat fed mice (HFD) [2,3]. Antibody neutralization of TGFβ action or inhibition of TGFβ expression in HFD-induced obese mice has been shown to ameliorate these phenotypes [4]. Cell culture studies in our laboratory had previously shown that CCN2 and TGFβ added in the first 48 h inhibited the differentiation process in 3T3L1 and primary fat cell cultures through inhibition of CEBPβ and CEBPδ along with the phosphorylation of Smad3 [5]. Several studies using 3T3L1 cells have shown that added TGFβ reduces the expression of UCP1 and its regulators PGC1α and PRDM16 thus playing a significant role in regulating the metabolic response of fat cells [2,6–8]. Other studies showed ALK7, one of the TGFβ receptors, was specifically expressed in mature adipocyte [9], and SMAD 6 and SMAD 7, modulators of TGFβ, were changed during adipocyte differentiation [10] suggessing that the there could be a different response to TGFβ depending upon the stage of adipocyte differentiation. This variable effect of TGFβ action has been observed in osteoblast where it has an inhibitory effect on differentiation at early stages of osteoblast differentiation whilst in mature osteoblasts it drove them to differentiate to osteoclasts [11]. But the literature is not clear if there would be a differential effect of exogenous TGFβ on transdifferentiation process between white and brown adipocyte characteristics depending on the stage of adipocyte differentiation. Furthermore, it is unclear if TGFβ regulates the PPARγ interaction with either TLE3 or PRDM16 that would largely determine their fate as white or brown like fat and related thermogenic capacity

The known pleotropic response to TGFβ in osteoblasts, coupled with the variations in the expression of TGFβ and its modulators (SMADS and Receptors) during different stages of adipocyte differentiation [9,10] prompted us to hypothesize that TGFβ could have different effects on thermogenic markers during the early stage of differentiation (commitment stage) or in the mature adipocyte (terminal differentiation stage). Therefore in our study we examined the effect of adding rhTGFβ to cells before (preadipocytes), during (commitment stages) and in the terminal differentiated state, on UCP1, its regulators: PGC1α [13], PRDM16 [14,15], UCP2, and on the adipogenic thermogenic markers PPARγ [16,17], TLE3 [18]. Difficulty in fully differentiating 3T3L1 cells has been noted in other studies, and rosiglitazone has been added to enhance adipocyte differentiation [19]. Because rosiglitazone itself stimulates PPARγ and may itself have effects on the white or beige fate of adipocyte, we used both the standard differentiation mix without rosiglitazone as well as the standard differentiation mix with rosiglitazone to account for any effect of rosiglitazone on TGFβ-induced changes in thermogenic markers or on other aspects of adipocyte differentiation.

## Materials and methods

### Culture and differentiation of 3T3L1 cells

3T3L1 cells (obtained from American Type Culture Collection) were cultured in six-well culture plates in Dulbecco’s Modified Eagle’s Medium containing 25 mM glucose (DMEM/high glucose), fetal calf serum (FCS, 10%) and penicillin/streptomycin (P/S, 1%) at 37°C with 5% CO_2._ Studies were commenced when cells were 95–100% confluence. A standard differentiation mix (S-Diff) containing 1 μg/ml insulin, 1 μmol/l dexamethasone and 115 μg/ml 3-isobutyl-1-methylxanthine (IBMX) was added or in a parallel series S-DIFF with rosiglitazone 1 μg/ml (R-Diff mix) was studied [19]. In all experiments, media were changed at day 3, 6 and 8 with standard growth media supplemented with insulin (1 μg/ml).

### TGFβ1 treatment

To study the adipogenic and thermogenic effect of rhTGFβ1, 3T3L1 cells at different phases of adipocyte differentiation (preadipocyte at commitment and at terminal differentiation stages) were treated with recombinant active human TGFβ1 protein (2 ng/ml: R& D Systems, Cat No 240-B-010). This dose has been shown previously and in the present study (data not shown) to up-regulate fibronectin gene expression by 6-fold compared with untreated cells [20].

The rhTGFβ1 (2 ng/ml) was added at day 0 to confluent fibroblasts (i) without differentiation mix (preadipocytes) or (ii) with S-diff or R-diff differentiation mix (commitment stage) and (iii) at day 10 to terminal differentiation stage with S-diff or R-diff differentiation mix all with 10% FCS. At day 10, the media were changed to DMEM containing 0.1% BSA and 12 h later the mature adipocytes were treated with rhTGFβ1 (2 ng/ml). Confluent un-differentiated 3T3L1 cells (Un-Diff) acted as control. For all studies, the media were removed 3 days after the addition of rhTGFβ1, the cells were washed twice with PBS (2 ml) prior to harvest for analysis of gene and protein markers for adipogenesis and thermogenesis.

### RNA extraction

The mRNA levels of markers for adipogenesis and thermogenesis were measured in cells harvested at the time points as described above. RNA was extracted from the cells using TRI reagent (Sigma). Briefly, 500 μl of TRI reagent was added to each well. The cells were then scraped from the plate and the mixture was transferred to eppendorf tubes. 1-Bromo-3-chlorpropane (100 μl) was added and samples incubated for 5–10 min at room temperature, prior to centrifugation at 12,000 ***g*** (Beckman Coulter, Allegra X-30R centrifuge) for 15 min at 4°C. The top layer was transferred to a 1.5 ml tube and 250 μl of isopropanol (99.5%) was added, the tubes were vortexed and placed at −20°C for up to 12 h. The RNA was then pelleted by centrifugation at 12,000 ***g*** for 15 min at 4°C. The pellet was washed by the addition of 500 μl of cold 70% ethanol and centrifugation at 12,000 ***g*** for 10 min at 4°C. The RNA pellet was resuspended in 20 μl of RNase free water, and the RNA quantity and purity were determined by measurement of the 260/280 ratio using the Nanodrop (Thermo Scientific), and for all samples, they were between 1.9 and 2.0. Total RNA was stored at −80°C for future use.

The RNA (1 μg) was reverse transcribed to cDNA using 50 pmol of oligo(dT)12-18 (Life Technologies) and 0.4 pmol of random hexamers (Life Technologies) in a PCR machine (Bio-Rad) using the following protocol: 10 min at 70°C, 10 mM of DTT (Life Technologies) and 0.05 mM of dNTPs (Bioline). Superscript l00 U (Life Technologies) was added and the samples incubated in a thermocycler for 10 min at 25°C, 60 min at 42°C, 10 min at 70°C and finally on hold at 4°C. The resulting cDNA was aliquoted to 384-well plates using the Freedom EVO-2 100 (Tecan) automated platform. After the addition of Sensi Mix™ SYBr® (Bioline) and 500 nM of forward and reverse primers (Table 1), the samples were amplified on a Lightcycler 480 (Roche) programmed for 10 min at 95°C, 40 cycles of 10 s at 95°C, 15 s at 60°C, 20 s at 72°C and then a final step held at 4°C. The mRNA levels were calculated using the Delta/Delta method, with NoNo used as the reference gene, qRT-PCR results were expressed as fold change relative to their respective controls.

**Table 1:** Lists the primers used in the present study

	Primers (mRNA)	Sequence	
**Adipogenesis Markers**	mus_PPARγ	Forward	5′-CTGTCGGTTTCAGAAGTGCCT-3′
		Reverse	5′-CCCAAACCTGATGGCATTGTGAGACA-3′
	mus_TLE3	Forward	5′-TTGTCACAGGAGCATCAGCAG-3′
		Reverse	5′-CAGATTGGGGAGTCCACGTA-3′
	mus_Pref1	Forward	5′-TTGTCACAGGAGCATCAGCAG-3′
		Reverse	5′-CAGATTGGGGAGTCCACGTA-3′
**Thermogenesis Markers**	mus_PGCα1	Forward	5′-CTGCGGGATGATGGAGACAG-3′
		Reverse	5′-TCGTTCGACCTGCGTAAAGT-3′
	mus_PRDM16	Forward	5′-TGACCATACCCGGAGGCATA-3′
		Reverse	5′-CTGACGAGGGTCCTGTGATG-3′
	mus_UCP1	Forward	5′-CATGGGATCAAACCCCGCTA-3′
		Reverse	5′-ATTAGGGGTCGTCCCTTTCC-3′
	mus_UCP2	Forward	5′-GGCCTCTGGAAAGGGACTTCT-3′
		Reverse	5′-TTGGCTTTCAGGAGAGTATCTTT-3′
**Reference Gene**	mus_NoNo	Forward	5′-TGCTCCTGTGCCACCTGGTACTC-3′
		Reverse	5′-CCGGAGCTGGACGGTTGAATGC-3′

The primers were designed using Primer-BLAST (Pubmed) (Table 1). All primers produced a single peak in melt curve analysis. Dilution curves for all genes were performed to establish the linear range and the qPCR efficiencies were between 90 and 110%.

### Protein extraction and quantification

The protein levels were measured by Western immunoblot in cells solubilized in RIPA buffer (100 μl) containing a cOmplete™ protease inhibitor cocktail (Roche). The cell lysate was collected in 1.5 ml eppendorf tubes and stored at −80°C.

Prior to analysis, the samples were thawed sonicated and then centrifuged at 12,000 ***g*** for 15 min at 4°C. The total protein content of the supernatant was determined using the DC™ Protein Assay (Bio-Rad). The sample containing 50 µg protein was mixed with loading buffer (laemmli loading buffer (4×) with dithiothreitol (50 mM): Sigma) and heated at 95°C for 10 min. Samples (15 μl) were then loaded onto (4-15%) Mini-PROTEAN TGX Stain-Free Precast Gradient Gels (4–15%: Bio-Rad). Gels were run at 130 V for 80 min. Samples were then transferred to PVDF membranes using Trans-Blot® Turbo™ Transfer System (Bio-Rad) and a standard turbo transfer protocol for 7 min. The membranes were incubated with 5% skim milk for 1 h and washed in TBST three times each for 10 min while shaken at room temperature. Membranes were incubated overnight at 4°C with primary antibody either Anti-UCP1 (Abcam, Cat No ab10983) or Anti-PRDM16 (Abcam, Cat No ab106410) at a 1:500 dilution in 1% skim milk, and membranes were washed in TBST three times prior to incubation with secondary antibody (1:10,000 dilution in 1% skim milk) labeled with peroxidase (Anti-rabbit IgG, Sigma®, Cat No S9169) for 1 h at room temperature. Excess antibody was removed by washing and the membranes were incubated in Clarity™ Western ECL Blotting substrate (Bio-Rad) prior to imaging using ChemiDoc™ MP System (Bio-Rad). Quantification of protein was undertaken by normalizing the size of the band of protein of interest against total protein load quantified using the stain free technique and ImageLab Software V4.1 (Bio-Rad) [21,22].

### Oil Red O staining

To track the differentiation of the 3T3L1 cells to fat cells, the cells were stained with Oil Red O (ORO) at day 10. For staining, the culture media were removed and wells were washed with 1 ml of PBS prior to fixation of the cells by the addition of 1 ml of 10% formalin for 30–60 min at room temperature. After removal of formalin, the cells were washed with 1 ml of PBS and incubated with 1 ml of 60% isopropanol for 5 min at room temperature. The isopropanol was then removed and cells were stained by the addition of 500 µl of ORO stain. After 5 min at room temperature, cells were rinsed twice with 1 ml of 60% isopropanol to remove excess stain. Images were obtained using an Olympus microscope at 10× objective.

### Statistical analysis

The data were analyzed using Prism Graph Pad 7 and the statistical differences were determined by one-way ANOVA with Tukey’s multiple comparison test, or where indicated, two-way ANOVA with Sidak’s multiple comparison test. One-way ANOVA was used to compare the effect of two differentiation mixes while two-way ANOVA was used to compare the effect of rhTGFβ1 on different differentiation time points expressed as mean ± SD with statistical significance being accepted at *P* < 0.05.

## Results

**Figure 1:**
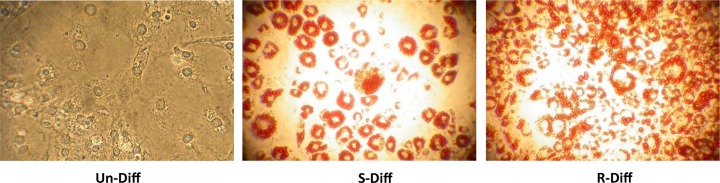
Effect of Diff mix on differentiation Picture taken at 100× magnification after Oil Red O staining on day 10 for undifferentiated (Un-Diff) and differentiated 3T3L1 adipocytes using S-Diff mix and R-Diff mix.

**Figure 2:**
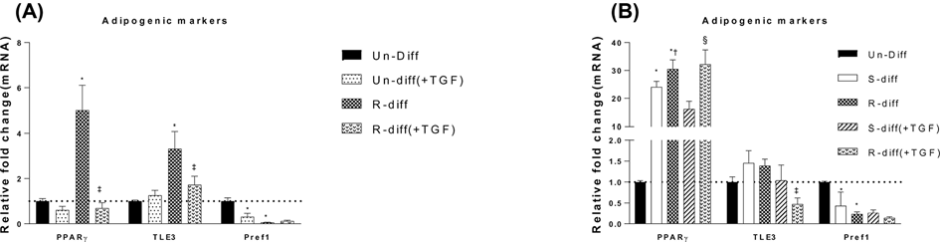
The effect of rhTGFβ1 on adipogenic marker mRNA levels of PPARγ, TLE3 and Pref1 during the different stages of adipocyte maturation (**A**) Un-Diff (preadipocytes, no differentiation mix) and early differentiated adipocytes at day 3. (**B**) Un-Diff (preadipocytes, no differentiation mix) and differentiated adipocytes at day 10. Results are the mean ± SD of triplicate samples in three independent experiments. Two-way ANOVA with Sidak’s multiple comparison test was used to compare the effect of rhTGFβ1 on Un-diff and S-diff during early differentiation shown in panel (A), and panel (B) shows S-Diff and R-Diff during late differentiation. One-way ANOVA with Tukey’s multiple comparison test was used to compare Un-Diff, S-Diff and R-Diff during late differentiation in panel (B). *P*-value<0.05 * versus Un-Diff; † versus S-Diff; ‡ versus R-Diff; § versus S-Diff (+TGF).

The differentiation of both R-Diff and S-Diff mixtures of preadipocytes to adipocytes at day 10 was demonstrated by the marked increase in their lipid staining with Oil Red O. Increased lipid staining was observed in cells with R-Diff compared with those with S-Diff adipocytes indicating a greater level of differentiation (Figure 1). This increased level of differentiation was further supported by the increased PPARγ seen in R-Diff 3T3L1 cells at both early (>5-fold at day 3) and late (>24-fold at day 13) phases of differentiation. In contrast both differentiation mixes had the same effect on Pref1 that is expressed at high levels in undifferentiated 3T3L1 cells (i.e. in a preadipocyte state), and was reduced by both differentiation mixes at both early (>100-fold) and late (>1-fold) phases of differentiation (Figure 2A,B). The expression of TLE3 that would drive the cells to a white adipose tissue rather than brown adipose tissue fate was also increased in adipocytes after the addition of the differentiation mixes (Figure 2A,B).

**Figure 3:**
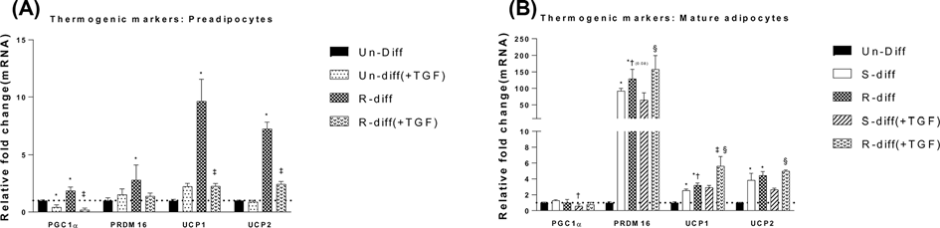
The adipogenic marker mRNA levels, PGC1α, PRDM16, UCP1 and UCP2 to rhTGFβ1 are dependent on the stage of adipocyte maturation Panel (**A**) compares Un-Diff (preadipocytes, no differentiation mix) and early differentiated adipocytes at day 3. Panel (**B**) compares Un-Diff (preadipocytes, no differentiation mix) and differentiated adipocytes at day 10. The results shown are mean ± SD of triplicate samples for three independent experiments. Two-way ANOVA with Sidak’s multiple comparison test was used to compare the effect of rhTGFβ1 on Un-diff and S-diff during early differentiation in panel (A) and for the comparison between S-Diff and R-Diff during late differentiation in panel (B). One-way ANOVA with Tukey’s multiple comparison test was used to compare Un-Diff, S-Diff and R-Diff during late differentiation in panel (B). *P*-value<0.05 * versus Un-Diff, † versus S-Diff. ‡ versus R-Diff, § versus S-Diff (+TGF).

Addition of rhTGFβ1 to preadipocytes down-regulated Pref1 and PGC1α, the genes regulating adipogenic and thermogenic responses (Figures 2A and 3A). Addition of rhTGFβ1 to R-diff treated cells at the early commitment stage of differentiation significantly down-regulated the gene expression of PPARγ (>7-fold), and this was not observed when rhTGFβ1 was added in the terminal differentiation stage (Figure 2A,B). rhTGFβ1 added to the R-Diff adipocytes decreased TLE3 at both commitment and terminal differentiation stages whereas it had no effect when added to S-Diff adipocytes at the terminal differentiation stage.

**Figure 4:**
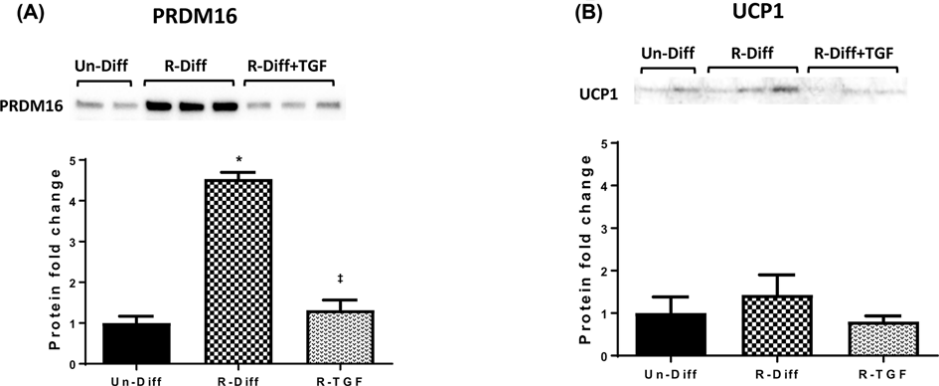
The effect of rhTGFβ1 on PRDM16 (A) and UCP1 (B) protein levels in early differentiated adipocytes (day 3) The Western blot is a representative result of three independent experiments and the graph is expressed as the mean ± SD. One-way ANOVA with Tukey's multiple comparison test was used to compare Un-Diff, R-Diff and R-TGF during early differentiation. *P*-value<0.05; *P*-value<0.05 * versus Un-Diff, ‡ versus R-Diff. Total protein loaded was obtained from stain free imaging and this was used as the loading control (figure provided in Supplementary Information).

**Figure 5:**
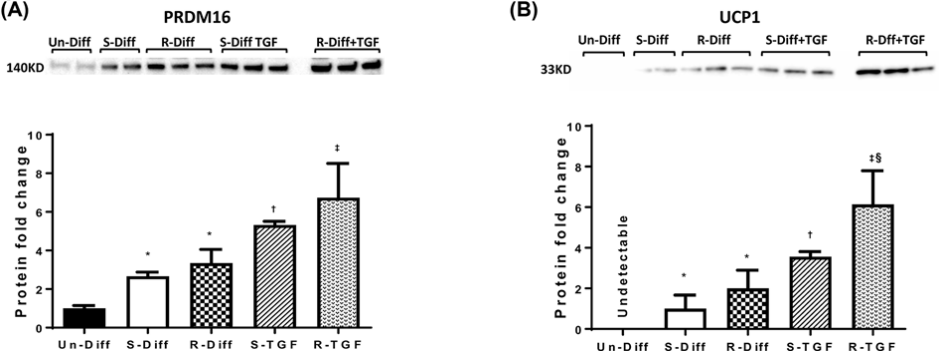
The effect of rhTGFβ1 on PRDM16 (A) and UCP1 (B) protein levels of mature adipocytes (day 10) The Western blot is a representative of the results from three independent experiments and the graph is expressed as mean ± SD. One-way ANOVA with Tukey’s multiple comparison test was used to compare Un-Diff, S-Diff and R-Diff. Two-way ANOVA with Sidak’s multiple comparison test used to compare the effect of TGFβ1 on S-Diff and R-Diff. *P*-value<0.05 * versus Un-Diff; † versus S-Diff; ‡ versus R-Diff; § versus S-Diff(+TGF). Total protein loading was obtained from stain free imaging and was used as the loading control (figure provided in Supplementary Information).

Investigation of the gene expression of adipocyte thermogenic markers at both commitment and terminal differentiation stage increased UCP1, PRDM16 and UCP2 (≥2-fold) (Figure 3A,B) and these changes were accompanied by increased levels of UCP1 and PRDM16 proteins (Figure 4A,B). Although an increase at the commitment stage of differentiation was observed in PGC1α mRNA (≥2-fold), it was not evident in the terminal differentiation stage (Figure 3A,B). Moreover, while the effect of R-Diff was greater in up-regulating the gene expression for UCP1 and PRDM16 (Figure 3B), there was only a small trend for increased UCP1 and PRDM16 protein expression (Figure 5A,B). RhTGFβ1 in the commitment stage of adipocytes significantly down-regulated the gene expression of PCG1α, UCP1 and UCP2 with indications for a trend in decreased PRDM16 (Figure 3A). Consistent with these changes in gene expression, rhTGFβ1 significantly down-regulated PRDM16 protein and there was a downward trend for the changes in UCP1 protein (Figure 4A,B). In the terminal differentiation stage of adipocytes, rhTGFβ1 did not show any significant changes in the gene expression of PRDM16 and UCP2 but did increase UCP1 mRNA and PRDM16 and UCP1 protein in R-Diff treated cells (Figure 5A,B). Curiously, PGC1α mRNA was consistently unchanged only in R-Diff when added at the terminal differentiation stage (Figure 3B).

## Discussion

The present study shows that in R-Diff cells, PPARγ is increased in commitment stage of adipocyte differentiation and the addition of rhTGFβ1 inhibited PPARγ, whereas both of the differentiation mixes decreased Pref1 (Figure 2). As could be predicted from other studies [23–26], we showed markedly increased mitochondrial biogenesis markers (PCG1α, PRDM16) that were accompanied by up-regulation in mitochondrial marker protein levels during adipocyte differentiation. Consistent with the increased PPARγ in R-Diff mix compared with S-Diff, our study showed that the thermogenic markers were also increased [27,28]. Furthermore, the increased PPARγ, PRDM16, UCP1 and decreased TLE3 in the present study are consistent with the proposed interaction of PRDM16 with PPARγ in stabilizing browning of adipocytes [29]. Interestingly, the change in thermogenic markers in our study and in others (13) was associated with small changes in PGC1α mRNA, but others have shown increases in PGC1β to compensate for the decreased PGC1α stimulation of mitochondrial biogenesis [28,30]. Collectively, the results reported herein and those of others suggested that independent of rosiglitazone presence in the differentiation mix, the induction of UCP1 during differentiation of adipocytes is not dependent on large changes in PGC1α in 3T3L1 cells. Also, PGC1α was consistently decreased at all three stages of adipocyte differentiation with rhTGFβ1 but it had a differential effect on UCP1 that depended on the phases of differentiation, suggesting that the induction of UCP1 in 3T3L1 cells by rhTGFβ1 in adipocyte may not be entirely dependent on PGC1α.

We found that the PRDM16 regulation of UCP1 in 3T3L1 adipocytes treated with rhTGFβ1 was dependent on the stage of differentiation. RhTGFβ1 down-regulated PRDM16 and UCP1 protein in the commitment stage of adipocytes but was up-regulatory in the terminal differentiation stage. A similar effect was seen on UCP2 mRNA in rhTGFβ1-treated cells (Figure 3A,B). This indicated that the effect of adding rhTGFβ1 to mature adipocytes may be metabolically protective and by favouring the beiging or browning of white adipocytes the increased UCP1 would enhance the futile cycle to burn excess calories [31]. Such an effect early in the commitment stage of differentiation may not be possible where TGFβ inhibited PRDM16 induction (Figures 3A and 4A). This effect in the commitment stage of adipocyte differentiation is consistent with known TGFβ effects on preventing proliferation and differentiation maintaining the cells in their preadipocyte state where the capacity for thermogenesis would not be required as shown by the decreased UCP1 (Figures 3A and 4B).

However, a whole body preclinical *in vivo* study done by Yadav et al showed that the inhibition of TGFβ signaling either by knocking down Smad3 or by using a TGFβ neutralizing antibody, increased UCP1 protein and mRNA [2]. They suggested that Smad3 pathways may have been responsible [32]. This observation corresponds to the effect of rhTGFβ1 on the commitment stage of adipocyte differentiation in our study. Thermoneutral cages for the animal studies have shown that the UCP1 response in WAT is different to that in animals stored at room temperature [33]. Our cell culture experiment equate to thermoneutral conditions and differences in the effect of TGFβ on thermogenic marker to that of Yadav et al. could be present. Other reports have also suggested that TGFβ increases lipid catabolism [34,35] and if it involved increased futile cycling then this could explain the up-regulation of thermogenic markers of mature adipocytes in our study. Since the effect of TGFβ differs depending on the stage of osteoblast maturity [11], a differential effect of TGFβ is also likely to be dependent on the adipocyte maturity. Although TGFβ receptors do not decrease in 3T3L1 cells during adipocyte differentiation, they do change the ratio of SMAD effector proteins and increase certain types of TGF family receptors [10]. These changes have been well documented in studies on mesenchymal differentiation and coupled with the known endogenous production of TGFβ suggest an autocrine role in adipocyte differentiation may be occurring [10]. Others have suggested that TGFβ can regulate the production of brite or beige progenitor cells in WAT [6–8]. Considering the pleotypic nature of the cellular responses to TGFβ, it is possible that the effect of rhTGFβ1 in the presence of increased PRDM16 in mature adipocytes could have different effects on UCP1, irrespective of which differentiation mix was used.

Villanueva et al. have shown that the white-selective cofactor, TLE3 acts reciprocally with PRDM16 (a brown-selective cofactor) to turn on lipid storage as opposed to increasing thermogenesis [18]. In our study, a consistent pattern was observed for the effect of rhTGFβ1 on thermogenic markers in mature adipocytes where increased PRDM16 was accompanied by increased UCP1 and a decreased TLE3. The same association was not observed in the rhTGFβ1 treatment given early in the differentiation or commitment process. This indicated that in mature adipocytes, the effect of rhTGFβ1 on thermogenesis could be by increasing the PRDM16 over TLE3 binding to PPARγ and would increase brown selective genes, an effect observed by others when TLE3 was deleted [18]. In addition to increasing thermogenesis, the increased PRDM16 in mature adipocytes would also reduce adipose tissue fibrosis and improve glucose homeostasis [12] by allowing adipose tissue to expand in response to the increased calorie load.

Hudak et al. have demonstrated that Pref1 maintains the preadipocyte phenotype [36]. Similarly, our study showed that gene expression of Pref1 was high in preadipocytes and significantly reduced in differentiated adipocytes (Figure 2). In undifferentiated preadipocytes, rhTGFβ1 was without effect on most of the adipogenic and thermogenic markers except for minimal effects on PGC1α and Pref1. However, once cells had fully differentiated, rhTGFβ1 bioactivity was observed that coincided with the expression of ALK7 TGF family receptors in adipocytes [9].

Overall, the results in the present work provide a comprehensive description of the effects of rhTGFβ1 upon the state of adipocyte maturation, with no beiging effect occurring in uncommitted preadipocytes and a robust beiging effect being realized in terminal differentiation stage treated with rhTGFβ1. The extent to which the cellular data relates to the *in vivo* situation will require further studies in animals and humans.

## Conclusion

There was a differential effect of rhTGFβ1 on the expression of thermogenic markers that was dependent upon the degree of adipocyte maturation. Shown by increased thermogenic markers in mature adipocytes, only minimal effects of rhTGFβ1 on thermogenic markers in the uncommitted preadipocytes and their poor response of thermogenic markers early in the differentiation process of adipocytes. These effects were associated with reciprocal changes in PRDM16, and TLE3 which in mature adipocytes resulted in increased UCP1 and UCP2.

## Supplementary Material

Supplementary Figure Information

## Abbreviations

FCS: fetal calf serum
IBMX: 3-isobutyl-1-methylxanthine
ORO: Oil Red O
R-Diff: Rosiglitazone differentiation mix
S-Diff: standard differentiation mix
TGFβ: transforming growth factor β
