# Supplementary material for: The effect of TGFβ1 on thermogenic markers is dependent on the degree of adipocyte differentiation
Source: Biosci Rep. 2020 May 14;40(5):BSR20194262. doi: 10.1042/BSR20194262 (PMC7225410; doi:10.1042/BSR20194262)
Supplement: Supplementary Figure Information [file BSR-2019-4262_supp.pdf]

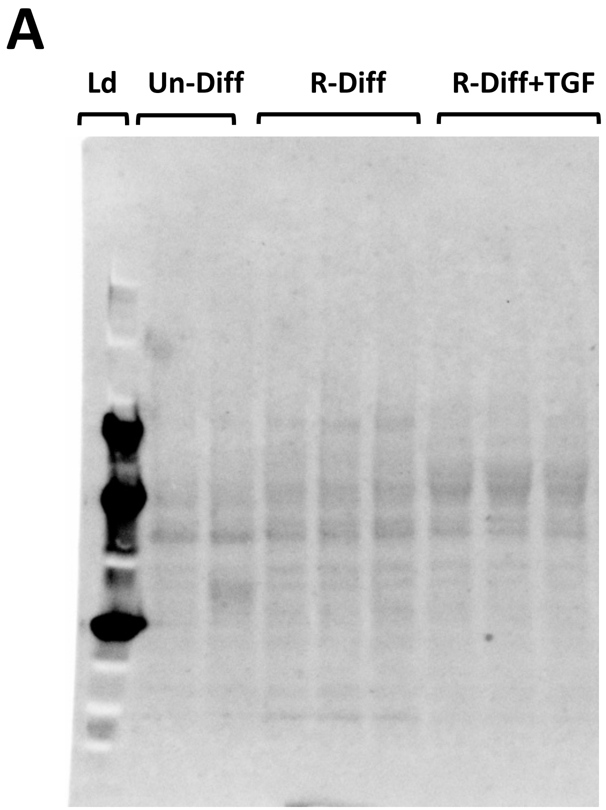

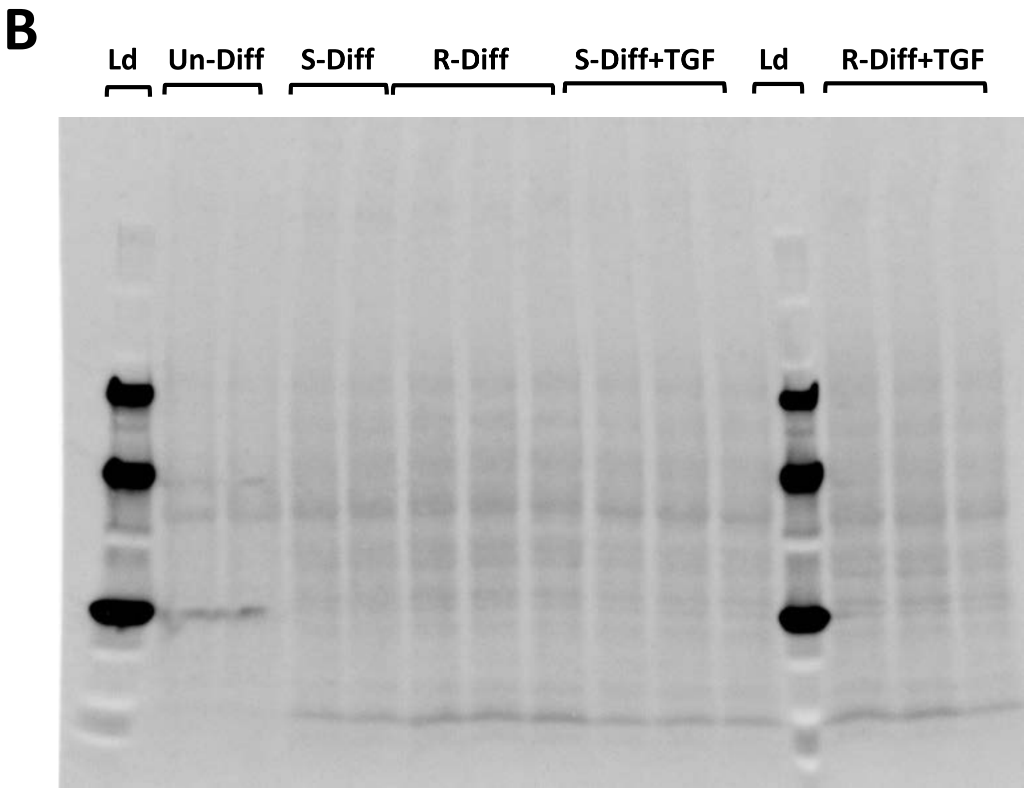

Supplementary Figure I

Total protein was obtained after running each gel using stain free imaging which provided the loading control for each lane.

Fig A: Representative gel for Un-Diff (preadipocytes, no differentiation mix) and early differentiated adipocytes at day 3 .

Fig B: Representative gel for Un-Diff (preadipocytes, no differentiation mix) and differentiated adipocytes at day 10.

Ld-indicates the molecular weight Ladder. Using this technique each lane acted as its loading control [18,19].
